# Minimally Invasive Diagnosis of Secondary Intracranial Lymphoma

**DOI:** 10.1155/2016/6165172

**Published:** 2016-11-28

**Authors:** A. P. McClement, G. M. Healy, C. E. Redmond, E. Stocker, G. Connaghan, S. J. Skehan, R. P. Killeen

**Affiliations:** ^1^School of Medicine, University College Dublin, Belfield, Dublin 4, D04 V1W8, Ireland; ^2^Department of Radiology, St. Vincent's University Hospital, Elm Park, Dublin 4, D04T6F4, Ireland; ^3^Department of Haematology, St. Vincent's University Hospital, Elm Park, Dublin 4, D04T6F4, Ireland

## Abstract

Diffuse large B cell lymphomas (DLBCL) are an aggressive group of non-Hodgkin lymphoid malignancies which have diverse presentation and can have high mortality. Central nervous system relapse is rare but has poor survival. We present the diagnosis of primary mandibular DLBCL and a unique minimally invasive diagnosis of secondary intracranial recurrence. This case highlights the manifold radiological contributions to the diagnosis and management of lymphoma.

## 1. Introduction

Diffuse large B cell lymphomas (DLBCL) are the most common form of non-Hodgkin lymphomas. While they usually present as a neck or abdominal nodal mass, they can arise in almost any extranodal site and are therefore frequently challenging to diagnose. Due to this varied presentation, radiology plays a central role in the diagnosis and management.

Early stage DLBCL is potentially curable. Survival for patients with more advanced disease varies widely depending upon multiple factors, including the genetic profile of the tumour [1]. Long term survival has been reported in up to 93% of patients with low risk disease [2]; however it is approximately 35% in those with the aggressive Activated B Cell (ABC) subtype [1]. The risk of central nerve system (CNS) relapse from an extracranial primary DLBCL ranges from 1.1 to 10.4% [3] and the median survival for these patients is 2–5 months [4]. Therefore, recognition of secondary CNS involvement is essential for accurate prognostication. Magnetic resonance imaging (MRI) is the primary modality used for the investigation of suspected cases; however, MRI appearances are not pathognomonic, necessitating histopathologic or cytologic samples to confirm diagnosis and exclude mimics (e.g., tuberculosis). Early diagnosis is paramount, in order to initiate and optimise therapy, but obtaining samples of intracranial lesions for histologic analysis can be challenging and risks iatrogenic neurological injury.

Here we report a case where the cytological diagnosis of intracranial DLBCL recurrence was achieved via a minimally invasive approach.

## 2. Case

A 65-year-old female presented to her dentist with throbbing left jaw pain and paresthesia of the left lip. She had a broken and infected lower left premolar tooth removed; however the paresthesia in the distribution of the left alveolar nerve persisted. At presentation she had a significant disability related to chronic obstructive pulmonary disease secondary to smoking (80-pack-year history) and she had a documented abdominal aortic aneurysm which was being monitored.

**Figure 1:**
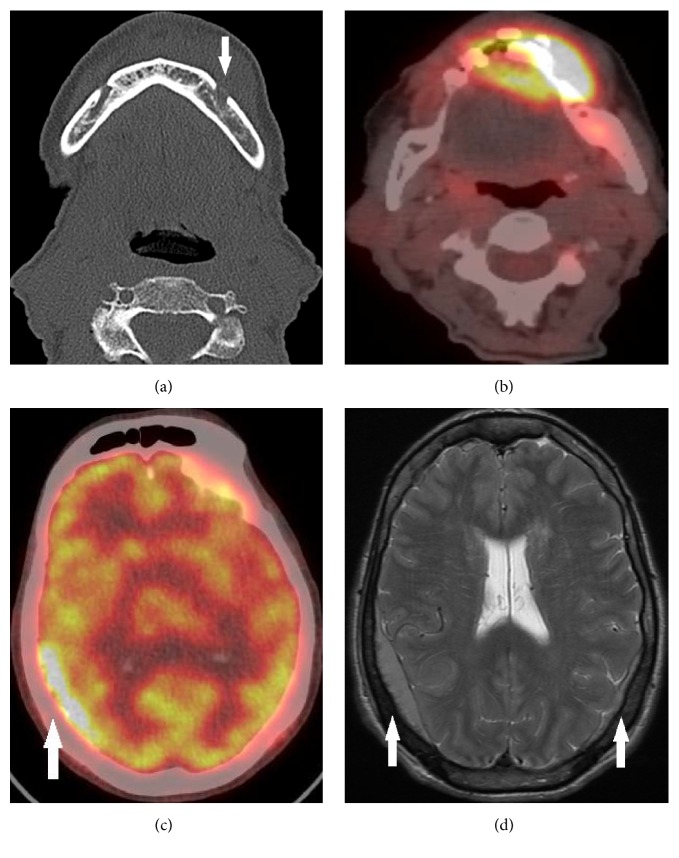
(a) CT of mandible, axial view, showing asymmetrical left alveolar canal widening (arrow); (b) PET CT showing uptake in the left mandible, left buccal mucosa, and left alveolar canal; (c) PET CT, axial view, showing increased uptake overlying the cortex of the right parietal lobe (arrow); (d) T2 weighted axial MRI of brain, showing presence of bilateral dural masses (arrows).

A noncontrast CT of the facial bones was performed for further assessment of her symptoms, which demonstrated sclerosis of the mandible in the midline with an overlying soft tissue mass and an asymmetric widening of the left alveolar canal. The latter finding raised the possibility of a malignant lesion with perineural spread (Figure 1(a)). An excisional biopsy was performed and histopathological analysis revealed diffuse large B cell lymphoma, ABC subtype. The tumour was positive for CD20, CD79a, Bcl-6, and MUM-1 (weak) but was negative for CD10, cyclin D1, Tdt, CD5, p53, and Bcl-2. Testing for c-myc and Epstein-Barr virus encoded RNA (EBER) was negative and the proliferative index was 90% as estimated by MIB-1. Fluorescence in situ hybridization (FISH) testing showed no translocations or rearrangements in myc, IgH t(8;14), Bcl-2, or Bcl-6. Lactate dehydrogenase was normal.

A baseline 18-fluorodeoxyglucose positron emission tomography (18-FDG-PET) scan identified abnormal tracer uptake (SUV max 19.6) in the left mandible and buccal mucosa. In addition, there was high tracer uptake along the left alveolar canal (Figure 1(b)), further suggestive of potential perineural tumour spread. There was no evidence of disease elsewhere. The patient suffered a non-ST elevation myocardial infarction during her work-up, requiring multivessel angioplasty and stenting of the right coronary and left circumflex vessels. Due to her advanced pulmonary and cardiac disease, the decision was made to treat her initially with 20 fractions of radiotherapy (total dose 40 Gy over 4 weeks) to her jaw, deferring potentially cardiotoxic chemotherapy until her cardiac status improved. She developed severe mucositis with ulceration after irradiation.

**Figure 2:**
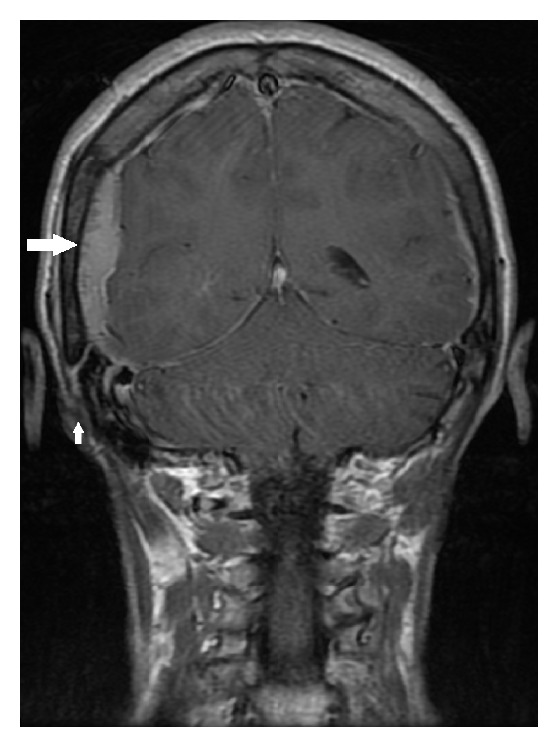
Postcontrast, T1 weighted coronal MRI of brain, showing extension of right side mass (large arrow) through the squamosal suture (small arrow).

A posttreatment repeat 18-FDG-PET, performed three months after completion of therapy, showed no tracer accumulation in the mandible, consistent with complete treatment response of the primary lesion. However, there were multiple new sites of uptake throughout the skeleton, in the right side of the neck and overlying the right posterior temporal and inferior parietal lobes of the brain (Figure 1(c)). An urgent MRI of brain was performed, showing bilateral enhancing dural masses overlying the parietal and occipital lobes (Figure 1(d)). On the right, the dural mass extended through the squamosal suture (Figure 2) into the postauricular scalp.

**Figure 3:**
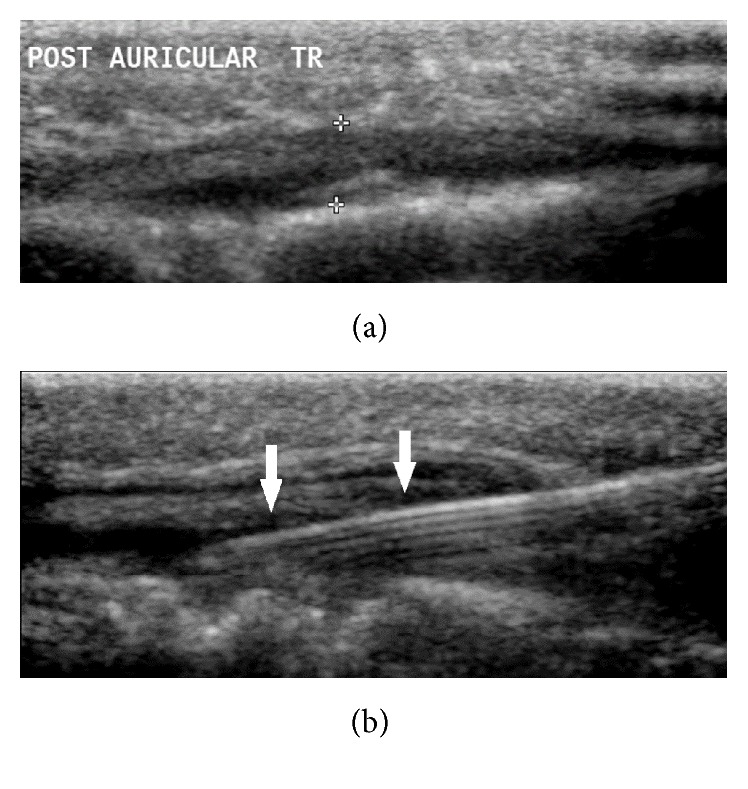
(a) Ultrasound imaging depicting the hypoechoic mass within the right postauricular soft tissues (cursors); (b) ultrasound guided FNA of extracranial extension of intracranial mass, with arrows indicating the needle with the lesion.

The scalp mass was then sampled using fine needle aspiration under direct ultrasound guidance (Figures 3(a) and 3(b)). Cytological analysis identified a population of single large atypical lymphoid cells, consistent with a recurrence of the patient's lymphoma. She proceeded to 20 fractions of whole brain radiotherapy (total dose 36 Gy) with subsequent R-CHOP (rituximab, cyclophosphamide, doxorubicin, vincristine, and prednisolone) plus lenalidomide chemotherapy. Initial chemotherapy was well tolerated but she presented just before course #3 with neutropenic sepsis with bilateral pneumonia. Despite aggressive therapy she succumbed to this infection.

## 3. Discussion

Primary non-Hodgkin's lymphoma of the mandible is rare, accounting for 0.6% of total cases [5]. It usually presents with jaw pain and can be difficult to differentiate clinically from conditions such as osteomyelitis, secondary neoplasms, or odontogenic lesions [6]. Other symptoms such as intraoral mass and tooth mobility may lead to misdiagnosis as a dental disorder [7–9]. More sinister signs, such as a nonhealing tooth socket, may prompt consideration for malignancy. Up to 20% of cases report paresthesia [6], and in this case the presence of subtle widening of the left alveolar canal raised the clinical suspicion of malignancy, since widening of this canal has been previously reported in lymphoma [10–13].

PET CT is an extremely important tool in the management of lymphoma, from initial diagnosis to assessment of treatment response and diagnosis of relapse. It is the primary modality used for staging this condition, where it detects approximately 20% more lesions than CT alone [14]. It can also be effective, when used in conjunction with MRI, in identifying perineural spread [15], as in this case. PET CT also plays a major role in evaluating response to therapy [16] and its role in detecting relapse is well established [17].

While CNS relapse of DLBCL is uncommon, it tends to occur early in the course of the treatment and patients with extranodal site involvement or immunodeficiency disorders are at higher risk [18]. Sutural involvement by intracranial non-Hodgkin's lymphoma has previously been documented [19]; however, to the best of our knowledge, direct extracranial extension has not been reported to date. Contrast enhanced MRI is the primary modality for investigating metastatic intracranial metastasis [20, 21]; however, definitive differentiation on MRI between CNS lymphoma and other causes of brain lesions is not possible [22]; therefore a histological diagnosis is required for confirmation.

The harvesting of a sample for histological analysis in such a case would usually require an open brain biopsy, with an associated risk of iatrogenic neurological sequelae. One study found a morbidity rate of 6% (mostly intracranial haemorrhage) and an overall mortality rate of 2.8% [23]. A retrospective study focusing on MRI guided stereotactic biopsy for intracranial lymphoma found a 4% mortality rate [24]. Alternative routes for diagnosis such as CSF flow cytometry and cytology are available; however they suffer from lower sensitivities of approximately 29.7% and 18.9%, respectively [25]. In addition, lumbar puncture would not be an option in the setting of potentially raised intracranial pressure. The minimally invasive sampling method used in this case therefore avoided the need for an open procedure and has, to our best knowledge, not been reported previously.

## 4. Conclusion

This is a unique case of minimally invasive diagnosis of secondary intracranial lymphoma, made possible due to direct transsutural growth of the tumour, thus avoiding requirement for invasive open biopsy.

## References

[1] Rosenwald A., Wright G., Chan W. C. (2002). The use of molecular profiling to predict survival after chemotherapy for diffuse large-B-cell lymphoma. *The New England Journal of Medicine*.

[2] Pfreundschuh M., Trümper L., Österborg A. (2006). CHOP-like chemotherapy plus rituximab versus CHOP-like chemotherapy alone in young patients with good-prognosis diffuse large-B-cell lymphoma: a randomised controlled trial by the MabThera International Trial (MInT) Group. *The Lancet Oncology*.

[3] Tai W. M., Chung J., Tang P. L. (2011). Central nervous system (CNS) relapse in diffuse large B cell lymphoma (DLBCL): pre- and post-rituximab. *Annals of Hematology*.

[4] Kridel R., Dietrich P.-Y. (2011). Prevention of CNS relapse in diffuse large B-cell lymphoma. *The Lancet Oncology*.

[5] Longo F., De Maria G., Esposito P., Califano L. (2004). Primary non-Hodgkin's lymphoma of the mandible. Report of a case. *International Journal of Oral and Maxillofacial Surgery*.

[6] Dinakar J., Priya L., Reddy S. (2010). Primary non-Hodgkin's lymphoma of the mandible. *Journal of Oral and Maxillofacial Pathology: JOMFP*.

[7] Djavanmardi L., Oprean N., Alantar A., Bousetta K., Princ G. (2008). Malignant non-Hodgkin's lymphoma (NHL) of the jaws: a review of 16 cases. *Journal of Cranio-Maxillofacial Surgery*.

[8] Panduric D. G., Kuna T., Katanec D. (2007). Pain after tooth extraction masking primary extranodal non-hodgkin's lymphoma of the oral cavity/Primarni ekstranodularni Non-Hodgkinov limfom usne supljine prikriven boli nakon vadenja zuba. *Acta Stomatologica Croatica*.

[9] Parrington S. J., Punnia-Moorthy A. (1999). Primary non-Hodgkin's lymphoma of the mandible presenting following tooth extraction. *British Dental Journal*.

[10] Bertolotto M., Cecchini G., Martinoli C., Perrone R., Garlaschi G. (1996). Primary lymphoma of the mandible with diffuse widening of the mandibular canal: report of a case. *European Radiology*.

[11] Yamada T., Kitagawa Y., Ogasawara T., Yamamoto S., Ishii Y., Urasaki Y. (2000). Enlargement of mandibular canal without hypesthesia caused by extranodal non-Hodgkin's lymphoma: a case report. *Oral Surgery, Oral Medicine, Oral Pathology, Oral Radiology, and Endodontology*.

[12] Mojaver Y. N., Sahebjamie M., Tirgary F., Eslami M., Rezvani G. (2005). Enlargement of mandibular canal with tongue paresthesia caused by extranodal B-cell Lymphoma: a case report. *Oral Oncology Extra*.

[13] Vartiainen V. M., Siponen M., Salo T., Rosberg J., Apaja-Sarkkinen M. (2008). Widening of the inferior alveolar canal: a case report with atypical lymphocytic infiltration of the nerve. *Oral Surgery, Oral Medicine, Oral Pathology, Oral Radiology and Endodontology*.

[14] Afshar-Oromieh A., Kratochwil C., Haberkorn U., Giesel F. L. (2012). Importance of PET/CT in lymphoma diagnostics. *Der Radiologe*.

[15] Paes F. M., Singer A. D., Checkver A. N., Palmquist R. A., De La Vega G., Sidani C. (2013). Perineural spread in head and neck malignancies: clinical significance and evaluation with 18F-FDG PET/CT. *Radiographics*.

[16] D'Souza M. D., Jaimini A., Bansal A. (2013). FDG-PET/CT in lymphoma. *Indian Journal of Radiology & Imaging*.

[17] Akkas B. E., Vural G. U. (2013). The incidence of secondary central nervous system involvement in patients with non-Hodgkin's lymphoma as detected by 1818F-FDG PET/CT. *Nuclear Medicine Communications*.

[18] Hill Q. A., Owen R. G. (2006). CNS prophylaxis in lymphoma: who to target and what therapy to use. *Blood Reviews*.

[19] Fest T., Rozenbaum A., Cattin F., Chambers R., Carbillet J.-P., Bonneville J.-F. (1988). Neuroblastoma-like epidural localization in non-Hodgkin's lymphoma. *Neuroradiology*.

[20] Maroldi R., Ambrosi C., Farina D. (2005). Metastatic disease of the brain: extra-axial metastases (skull, dura, leptomeningeal) and tumour spread. *European Radiology*.

[21] DeAngelis L. M., Boutros D. (2005). Leptomeningeal metastasis. *Cancer Investigation*.

[22] Haldorsen I. S., Espeland A., Larsson E.-M. (2011). Central nervous system lymphoma: characteristic findings on traditional and advanced imaging. *American Journal of Neuroradiology*.

[23] Malone H., Yang J., Hershman D. L., Wright J. D., Bruce J. N., Neugut A. I. (2015). Complications following stereotactic needle biopsy of intracranial tumors. *World Neurosurgery*.

[24] Malikova H., Liscak R., Latnerova I., Guseynova K., Syrucek M., Pytlik R. (2014). Complications of MRI-guided stereotactic biopsy of brain lymphoma. *Neuro Endocrinology Letters*.

[25] Schroers R., Baraniskin A., Heute C. (2010). Diagnosis of leptomeningeal disease in diffuse large B-cell lymphomas of the central nervous system by flow cytometry and cytopathology. *European Journal of Haematology*.

